# Supplementary material for: Magnetic reconnection driven by electron dynamics
Source: Nat Commun. 2018 Nov 30;9:5109. doi: 10.1038/s41467-018-07415-3 (PMC6269529; doi:10.1038/s41467-018-07415-3)
Supplement: Supplementary file 1 — Supplementary Information [file 41467_2018_7415_MOESM1_ESM.pdf]

Supplementary information

Magnetic reconnection driven by electron dynamics

Kuramitsu et al.

Supplementary Method: Particle-in-cell simulations

We perform particle-in-cell (PIC) simulations in two spatial dimensions (2D) in order to support our model described in the paper. Since the information obtained from the experiments is limited, we still need the numerical support to understand the details of the phenomena (For instance, in space plasmas, there is no global observation, and thus, the numerical simulations can provide the global information as in [1]). We inject plasma along the x axis of the simulation system where no background plasma exists. Electrons and ions are injected at the same position in each computational time step so that plasma density n_0 is approximately constant there. The injection velocity is larger than the thermal speed of ions $v_0/v_{ti} = 25$ and electrons $v_0/v_{te} = 2.5$, where v_0, v_{ti}, and v_{te} represent the plasma injection velocity and the thermal velocities of ions and electrons, respectively. We assume the same temperature for ions and electrons $T_i = T_e$. We apply a weak-uniform magnetic field B_0 in the direction perpendicular to the plasma injection, in the $+y$ direction on the simulation plane. The typical β_K for electrons and ions are ~ 40 and ~ 4000, respectively. We set the mass ratio as $m_{\mathrm{i}}/m_{\mathrm{e}} = 100$ due to the limited computational resources. The ratio between the electron plasma frequency and the electron gyro frequency $\omega_{\mathrm{pe}}/\omega_{\mathrm{ce}}$ is typically ~ 18. Here, β_K and $\omega_{\mathrm{pe}}/\omega_{\mathrm{ce}}$ are defined with v, n_0 and B_0. The number of particles per cell is $\sim$ 1920 for each species at the injection point. The number of mesh is $(N_x, N_y) = (4096, 4096)$ and the sizes of the grid and the system are $\Delta x = \Delta y = 0.06 r_{\mathrm{ge}}$ and $L_x = L_y = 2.5 r_{\mathrm{gi}}$ where r_{ge} and r_{gi} are the typical electron and ion gyroradii defined with v_0 and B_0. The time step $\Delta t \omega_{\mathrm{ce}} = 0.0075$. For the x-direction, the radiation boundary condition is employed for electromagnetic fields and the particles reached the boundary are simply discarded. Periodic boundary condition is employed for the y-direction. For comparison, we perform an additional simulation without background magnetic field, keeping the other physical conditions.

Supplementary Figures 1 (a) and 1 (b) show 2D images of the electron (upper) and charge (lower) density with and without the magnetic field, respectively. The curved lines

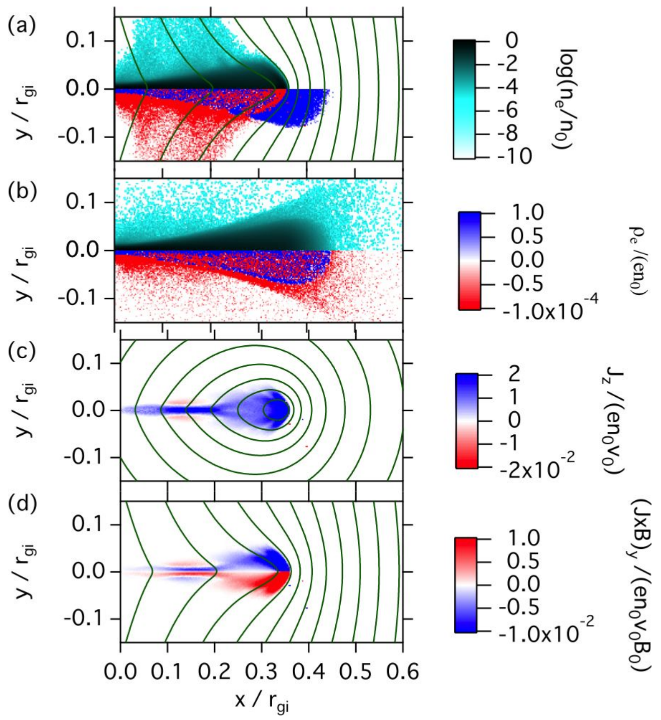

Supplementary Figure 1: **Particle-in-cell simulations.** (a) Under influence of the background magnetic field, electron density n_e normalized to n_0 and the charge density normalized to en_0 are shown in upper and lower panels, respectively. (b) Same as (a) but no background magnetic field. (c) The z component of the current density J_z normalized to en_0v_0. (d) The y component of the $\mathbf{J} \times \mathbf{B}$ force normalized to $en_0v_0B_0$. Curved lines in (a) and (d) indicate the field lines of the total magnetic field (initial field + induced field) while those in (c) indicate the field lines of the induced magnetic field by the current. The magnetic field is upward direction in (a) and (d), and counterclockwise direction in (d). These profiles are obtained at $t\omega_{\rm ci} = 1.13$.

in Supplementary Figure 1 (a) represent the magnetic field lines. Since the ion gyroradius is much larger than the system size, only electrons are magnetized and trapped by the magnetic field. The positive charge ahead of the electrons are from ions. This creates a space charge and a strong electrostatic field. In the absence of the background magnetic field electrons simply follow the ions, and thus, there is no such space charge in Supplementary Figure 1 (b). Only in the presence of the magnetic field, the electrons are concentrated on the propagation axis of the plasma. The electrons perform the $\mathbf{E} \times \mathbf{B}$ motion in the $-z$ direction and create the finite current in Supplementary Figure 1 (c). The curved lines in Supplementary Figure 1 (c) represent the magnetic field lines included by this current.

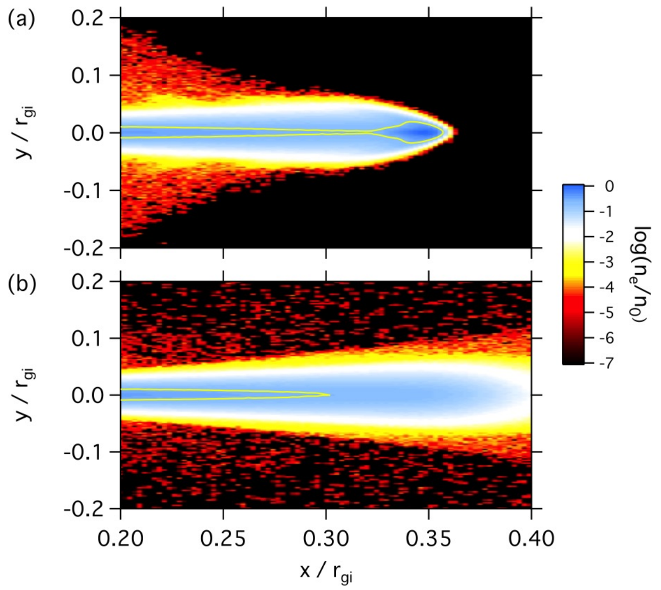

Supplementary Figure 2: **Density with contours.** (a) Magnified view of the electron density in the presence of the external magnetic field, shown in the upper half of Fig. 1 (a). The contour line shows a certain density. (b) Same as (a) but corresponding to the Fig.. 1 (b).

Supplementary Figure 1 (d) shows the y component of the $\mathbf{J} \times \mathbf{B}$ force and the curved lines represent the total magnetic field lines, as the same as Supplementary Figure 1 (a). As evidently seen, the plasma is further collimated where the magnetic field is distorted.

Supplementary Figures 2 (a) and 2 (b) show the magnified views of the upper halves (electron density) of Supplementary Figures 1 (a) and 1 (b) with different color and the contour lines at the same density. With interferometry we can observe the density higher than the detectable threshold determined by the resolution of imaging system. Suppose that the detectable density corresponds to the contour lines, the plasma with the external magnetic field extends further than that without the magnetic field. In Fig. 1 (e) if we could collimate the plasma, the detectable region was extended as in Fig. 1 (f). There is certainly a possible explanation why the plasma extends further in the presence of the external magnetic field without assuming the plasma velocity is faster.

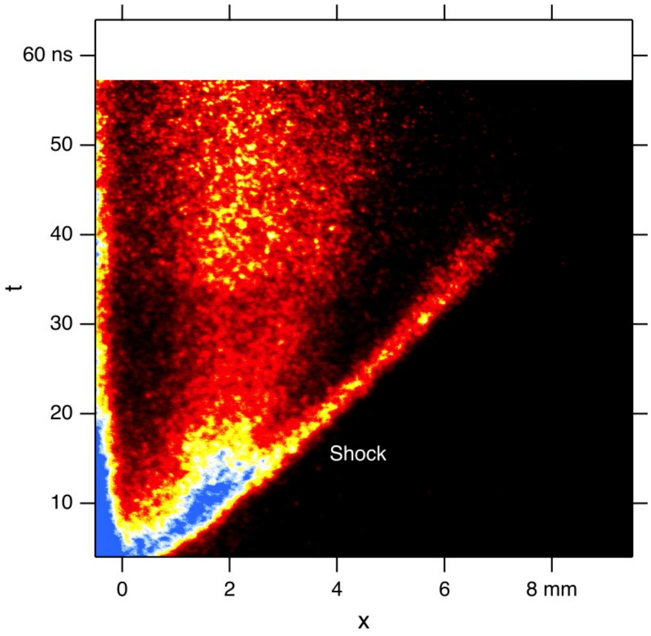

Supplementary Figure 3: **Time evolution of the plasma in the absence of the external magnetic field.** An SOP image similar to Fig. 4 (b) except there is no external magnetic field.

Supplementary Figure: Self-emission image in the absence of the external magnetic field

In Supplementary Figure 3 we show a similar image to Fig. 4 (b) except there is no external magnetic field. The shock velocity is slightly faster in Supplementary Figure 3 than that in Fig. 4 (b). This clearly shows that the plasma velocity in the presence of the external magnetic field is similar to or slower than that in the absence of the magnetic field.

In Supplementary Figure 3 there is no signal after 57 ns since the triger timing is 14 ns earlier than that of Fig. 4 (b) to confirm the laser timing (t = 0 ns, not shown). One can see lower sensitivity of the camera from ~ 20 to ~ 35 ns in Supplementary Figure 3, which corresponds to ~ 34 to ~ 49 ns in Fig. 4 (b). The lower sensitivity is due to the fact that we use the central region of CCD for the alignment of the optical system. These are technical details and nothing to do with our model and interpretation. We would like to emphasize that the plasmoid like feature is in this less sensitive region in Fig. 4 (b) and there is no plasmon like feature in Supplementary Figure 3.

Supplementary References

---

[1] Burch J. L. et al., `Electron-scalemeasurementsofmagneticreconnectioninspace`, Sci. 352, aaf2939 (2016).
